# Conservative management of primary vaginal endodermal sinus tumor and rhabdomyosarcoma

**DOI:** 10.18632/oncotarget.18829

**Published:** 2017-06-28

**Authors:** Weimin Xie, Keng Shen, Jiaxin Yang, Dongyan Cao, Mei Yu, Yao Wang

**Affiliations:** ^1^ Department of Obstetrics and Gynecology, Peking Union Medical College Hospital, Chinese Academy of Medical Sciences and Peking Union Medical College, Beijing 100730, China

**Keywords:** vaginal malignancies, endodermal sinus tumor, rhabdomyosarcoma, children, prognosis

## Abstract

The aim of this study was to evaluate the conservative management and prognosis of primary vaginal endodermal sinus tumor and rhabdomyosarcoma in children. Medical records of children with vaginal endodermal sinus tumor and rhabdomyosarcoma between 1996 and 2015 were reviewed. A total of 24 patients (median age, 12 months; range, 7–44 months) were included in this study, comprising 17 patients with endodermal sinus tumor and 7 patients with rhabdomyosarcoma. Among the 17 patients with endodermal sinus tumor, 15 were initially treated at our hospital with chemotherapy alone, and 2 were initially treated in other hospitals with conservative surgery and chemotherapy. All 7 patients with botryoid rhabdomyosarcoma received chemotherapy without well-defined protocols. At a median follow-up of 51 months (range, 4–237 months), 3 patients (12.5%; 1 with endodermal sinus tumor and 2 with rhabdomyosarcoma) developed recurrence. At the last follow-up, 22 patients (91.7%) were alive without evidence of disease, 1 patient with botryoid rhabdomyosarcoma died of disease progression, and 1 patient with endodermal sinus tumor died of respiratory and circulatory failure. To allow preservation of sexual and reproductive function, conservative therapeutic strategies should be considered for children with vaginal endodermal sinus tumor and botryoid rhabdomyosarcoma.

## INTRODUCTION

Primary vaginal malignancies are rare tumors of the female genital system, accounting for approximately 0.3% of all invasive cancers among women, and 1%–2% of all gynecologic malignant neoplasms [1]. In children, vaginal malignancies usually present with abnormal vaginal bleeding, blood-tinged discharge, tissue protruding from the vagina, and abdominal pain [2–4].

Rhabdomyosarcoma (RMS) is the most common vaginal malignancy in childhood, followed by malignant germ cell tumors (MGCTs) and clear cell adenocarcinoma [3–4]. RMS is the most common soft-tissue sarcoma in childhood, which usually occurs in the head and neck, followed by the genitourinary tract [5]. The newly described International Classification of Rhabdomyosarcoma (ICR) reported by the Intergroup Rhabdomyosarcoma Study Group (IRSG) recognizes three different subtypes: embryonal, alveolar, and undifferentiated [6, 7]. The embryonal subtype can be subdivided to classic, botryoid, and spindle cell variants [6, 7]. Among these subtypes, botryoid RMS mainly occurs in the vagina during infancy and early childhood (mean age, 3 years) [8–9]. Vaginal MGCTs are relatively rare tumors; endodermal sinus tumor (EST) is the most common histological subtype that occurs primarily in infants [10].

Owing to the rarity of primary vaginal EST and RMS in childhood, there is sparse literature on this subject, and a consensus concerning standard treatment protocols is still lacking. This study aimed to evaluate the conservative management and prognosis of primary vaginal EST and RMS.

## RESULTS

A total of 24 patients (EST, n = 17; RMS, n =7) were included in this study. The age of patients at diagnosis ranged from 7 to 44 months (mean: 17.1 months, median: 12 months). No patients had documented prenatal exposure to diethylstilbestrol or oral contraception.

### Endodermal sinus tumor

Clinicopathologic data of the 17 patients with EST are listed in Table 1. The age of patients ranged from 7 to 44 months (mean: 15.4 months, median: 11 months). All 17 patients presented with vaginal bleeding as their first symptom; 4 also had complaints of a protruding mass. The greatest tumor diameter ranged from 1 to 7 cm (mean: 4.1 cm, median: 4.0 cm). Serum alpha-fetoprotein (AFP) levels were markedly elevated in all patients (range, 250.6–54,000 ng/ml).

**Table 1:** Clinicopathologic features of 17 cases with vaginal endodermal sinus tumor

Case	Age (months)	AFP level (ng/ml)	Tumor size (cm)	Initial treatment		Cycles before CR	Recurrence	Status (months)
				Surgery	Chemotherapy (cycles)			
1	20	15,100	3	–	PEB (3) + PEV (5) + VAC (1)	3	–	NED (237)
2	44	646.14	5	–	PEB (5) + PEV (1) + VAC (1)	2	–	NED (234)
3	10	13,890	5	–	PEB (5)	3	–	NED (162)
4	11	10,268	6	–	PEB (6)	3	–	NED (78)
5	13	768.2	2	–	PEB (4)	2	–	NED (74)
6	11	1,110	6	–	PEB (5)	2	–	NED (67)
7	8	4,638	4	–	PEB (4)	2	–	NED (56)
8	10	8,754	4	–	PEB (1) + PEV (3) + VAC (1)	2	–	NED (52)
9	7	29,987	6	–	PEB (5)	3	–	NED (50)
10*	36	54,000	7	Resection	PEB (10)	3	–	NED (78)
11	9	364.6	1	–	PEB (4)	3	–	NED (36)
12	11	1,289	4	–	PEB (4)	2	–	NED (27)
13	28	250.6	1	–	PEB (4)	2	–	NED (28)
14	9	19,766	6	–	PEB (6)	4	–	NED (25)
15	8	2,626	2	–	PEB (4)	3	–	Dead (4)
16*	15	253	4.5	Resection + L + IIVE	NVBE (1) + PEB (2)	1	–	NED (18)
17	11	3,293	3	–	PEB (5)	3	Vagina, 7 months	NED (12)

EL, exploratory laparotomy; L, lymphadenectomy; IIVE, internal iliac venous embolectomy; PEB, cisplatinn, etoposide, bleomycin; PEV, cisplatinn, etoposide, vincristine; VAC; vincristine, actinomycin, cyclophosphamide; NVBE, nedaplatin, vindesine, bleomycin, etoposide; NED, no evidence of disease.

* Cases who were initially treated in other hospitals.

Fifteen patients were initially diagnosed and treated in our hospital. Fourteen patients underwent examination and were histopathologically diagnosed by vaginal biopsies. One patient was diagnosed based on a mass discharged from the vagina. Following the definitive diagnosis, all 15 patients immediately received cisplatin, etoposide, and bleomycin (PEB) chemotherapy. The first 6 patients were treated with a conventional dosage of PEB, which consisted of cisplatin 100 mg/m^2^ per cycle (30∼35 mg/m^2^ on days 1 to 3) every 3 weeks, etoposide 100 mg/m^2^ on days 1 to 3 every 3 weeks, and bleomycin 15 mg/m^2^ once per week. The remaining 9 patients were administered a reduced dosage of bleomycin 15 mg/m^2^ on days 1 and 2 every 3 weeks. Owing to inexperience with regard to side effects in infants, the chemotherapy protocols of the first 2 patients were changed from PEB to cisplatin, etoposide, and vincristine (PEV), and vincristine, actinomycin, and cyclophosphamide (VAC), to prevent potential lung toxicity due to bleomycin. Serum AFP levels were monitored before every cycle to evaluate tumor response. Chemotherapy was considered effective if the serum AFP level decreased logarithmically every cycle. The average number of cycles until the AFP level decreased to a normal range was 2.6 (range 2–4). The patients received a mean of 5.1 cycles (range 4–9) of chemotherapy before completing treatment. Two patients were initially treated at other hospitals and later referred to our hospital. Case 10 underwent vaginal tumorectomy via laparotomy, and received 10 cycles (repeated every 3 weeks) of PEB (cisplatin 20 mg/m^2^ on days 1 to 5, etoposide 100 mg/m^2^ on days 1 to 5, and bleomycin 15 mg/m^2^ on day 1). Case 16 underwent vaginal tumorectomy plus pelvic lymphadenectomy and internal iliac venous embolectomy via laparotomy. The histopathologic results revealed tumor involvement in the internal iliac vein; however, all pelvic lymph nodes were negative. She received 1 cycle of nedaplatin, vindesine, bleomycin, and etoposide chemotherapy and was then referred to our hospital. After routine evaluation, we changed the treatment protocol to PEB.

After the AFP level decreased to a normal range, all the patients were evaluated with diagnostic imaging, such as magnetic resonance imagining (MRI) and computed tomography (CT), and examination under anesthesia. In total, 14 patients with suspected residual lesions underwent vaginal biopsies; all lesions were confirmed negative for malignancy. All 17 (100%) patients achieved complete remission. The most common chemotherapeutic side effects included bone marrow suppression, nausea, and vomiting; 1 patient (5.9%, 1/17) developed interstitial lung disease after 1 cycle of PEB chemotherapy and then changed to PEV and VAC chemotherapy.

The median follow-up duration was 52 months (range, 4–237 months). Case 15 developed acute dyspnea and heart failure following severe vomiting, and subsequently died of respiratory and circulatory failure. Only 1 patient (5.9%, 1/17) developed vaginal recurrence at 7 months after diagnosis. She received 5 cycles of PEB and achieved complete remission again. At the last follow-up, 16 patients (94.1%, 16/17) were alive without evidence of disease.

### Rhabdomyosarcoma

Clinicopathologic data of the 7 patients with RMS are listed in Table 2. The age of the patients ranged from 8 to 39 months (mean: 21.4 months, median: 23.0 months). At presentation, a protruding mass was found in 6 patients, and vaginal bleeding following by a protruding mass was found in 1. The greatest tumor diameter ranged from 2 to 7 cm (mean: 4.9 cm, median: 5 cm). According to the ICR classification, all 7 patients were diagnosed with botryoid RMS. According to the IRSG surgically based clinical grouping system, 1 patient had a Group I tumor, and 6 had Group III tumors.

**Table 2:** Clinicopathologic features of 7 cases with vaginal rhabdomyosarcoma

Case	Age (months)	Histology	Group	Tumor size (cm)	Initial treatment		Site(s) of Recurrence	Time to Recurrence (months)	Status (months)
					Surgery	Chemotherapy (cycles)			
18	39	B-RMS	III	7	Excisional biopsy	PVB (1) + VE (3) + VACP (3)	Inguinal LN	15	DOD (28)
19	16	B-RMS	III	3	Excisional biopsy	IVA (4) + VCE (2)	–	–	NED (102)
20	26	B-RMS	III	3	Excisional biopsy	VAC (12)	–	–	NED (84)
21	13	B-RMS	III	7	Excisional biopsy	IVA (4) + VCE (2)	–	–	NED (58)
22	8	B-RMS	III	7	Excisional biopsy	IVA (4)	Inguinal LN	10	NED (46)
23	25	B-RMS	III	5	Excisional biopsy	IVA (4)	–	–	NED (32)
24	23	B-RMS	I	2	Local excision	IVA (2)	–	–	NED (23)

B-RMS, botryoid rhabdomyosarcoma; PVB, cisplatin, vincristine, bleomycin; VE, vincristine, etoposide; VACP, vincristine, actinomycin, cyclophosphamide, cisplatin; IVA, ifosfamide, vincristine, actinomycin; VCE, vincristine, carboplatin, etoposide; VAC, vincristine, actinomycin, cyclophosphamide; LN, lymph nodes; DOD, dead of disease; NED, no evidence of disease.

Six patients underwent examination under anesthesia and excisional biopsy; 1 patient whose tumor had a slender pedicle underwent complete local excision. Following the histopathologic diagnosis, all patients received chemotherapy for the initial treatment without well-defined protocols. The first patient (case 18) received sequential cisplatin, vincristine, and bleomycin; vincristine and etoposide; and vincristine, actinomycin, cyclophosphamide, and cisplatin chemotherapy. Five patients received ifosfamide, vincristine, and actinomycin (IVA) based on the results of the third International Society of Paediatric Oncology (SIOP) study [11]. IVA was comprised of ifosfamide 3 g/m^2^ on days 1-3 (with mesna and hydration); vincristine 1.5 mg/m^2^ (maximum dose of 2 mg) on day 1 (weekly for weeks 1-6); and actinomycin on 1.5 mg/m^2^ (maximum dose of 2 mg) on day 1; repeated every 3 weeks. Among these, the regimens of 2 patients who showed a poor response (<50% partial response) at the first evaluation were changed to vincristine, carboplatin, and etoposide (VCE) chemotherapy. VCE was comprised of vincristine 1.5 mg/m^2^ (maximum dose of 2 mg) day 1; carboplatin 600 mg/m^2^ on day 1; and etoposide 150 mg/m^2^ on day 1; repeated every 3 weeks. One patient received VAC chemotherapy according to the IRSG recommendation. The patients received a mean of 5.9 cycles (range 2–12) of chemotherapy before completing treatment. During chemotherapy, all 7 patients underwent a second evaluation with examination under anesthesia. Five patients underwent resection of residual tumors, with the histopathologic results revealing complete necrosis in 4 patients, and inflammatory tissue in 1 patient. Two patients who had no residual tumor also underwent tumor site biopsy, and the histopathologic results confirmed negativity for RMS. The most common chemotherapeutic side effects included bone marrow suppression, nausea, vomiting, and hair loss.

The median follow-up duration was 46 months (range, 23–102 months). Two (28.6%) of the 7 patients developed metastatic recurrence localized to the inguinal lymph nodes at 10 and 15 months after diagnosis, respectively. Both of them underwent inguinal lymphadenectomy, and lymph node involvement was confirmed on histopathology. One received an intensified six-drug chemotherapy including IVA, carboplatin, epirubicin, and vincristine; and ifosfamide vincristine, and etoposide based on the results of the third SIOP study [11]. The other patient decline further therapy and died of tumor progression. At the last follow-up, 6 patients (85.7%, 6/7) were alive without evidence of disease.

## DISCUSSION

Historically, treatment protocols for primary vaginal EST and RMS are very aggressive but have a poor prognosis, such as radical surgery (ranging from vaginectomy to total pelvic exenteration), and external radiation or vaginal brachytherapy, leading to a loss of reproductive function [4, 12]. Recently, the development of chemotherapy has permitted a conservative strategy to maintain sexual and reproductive function for the future [10, 12]. Owing to the rarity of these tumors, the literature provides limited data on conservative therapeutic strategies, and most of the current experience is derived from case reports. To our knowledge, these 24 cases represent the largest series of primary vaginal EST and RMS in childhood in the literature.

Vaginal EST is an uncommon MGCT that almost exclusively occurs in children younger than 3 years old. Patients typically present with vaginal bleeding, with or without a polypoid mass protruding from the vagina [12, 13]. Serum AFP has been considered a reliable marker for diagnosis, evaluation of treatment response, and surveillance for recurrence. In our series, the AFP level was markedly elevated before treatment, and dramatically decreased after chemotherapy in all 17 patients. The patient who developed vaginal recurrence was first detected according to the elevated AFP level.

Vaginal EST is a highly malignant tumor that is both locally aggressive and capable of metastasizing via hematogenous and lymphatic pathways [14]. Before 1965, local therapeutic modalities (aggressive surgery and/or irradiation) were used exclusively, with universal failure [15]. In one series of 32 patients with vaginal EST, 18 (56%) died of the disease despite radical surgery [16]. Since the 1970s, various combination chemotherapy regimens have been introduced and have improved survival significantly [14]. Among these, platinum-based chemotherapy has been one of the most commonly used regimens [10]. PEB has been the most acceptable and successful chemotherapy in treating vaginal EST [12, 17]. Chemotherapy alone should be considered for treating vaginal EST to maintain sexual and reproductive function [10, 17–20]. Limited cases have been successfully treated with PEB chemotherapy alone [10, 13, 17–20] (Table 3). Our previous study that reported our first 6 patients with vaginal EST also showed excellent outcomes with PEB chemotherapy alone [21]. In the present study, 15 patients were treated with chemotherapy alone; of these, 12 received PEB chemotherapy alone, and 3 received combinations of PEB, PEV, and VAC chemotherapy. As a result, all 15 patients achieved complete remission. Two patients who underwent conservative surgery also had excellent responses to PEB chemotherapy. Previous studies [17, 18, 20], in addition to our own experiences, have indicated that PEB chemotherapy alone could be the first choice of treatment for patients with vaginal EST.

**Table 3:** Results of PEB chemotherapy alone for vaginal endodermal sinus tumor in the literature

References	Case	Age (months)	AFP level (ng/ml)	Chemotherapy (cycles)	Follow-up, results
Handel et al. [10]	1	6	8,913	PEB (N/A)	NED (3 mo)
	2	15	24	PEB (N/A)	NED (84 mo)
	3	8	6,913	PEB (3)	Local recurrence 4 mo later, NED (78 mo)
Davidoff et al. [13]	1	Mean age 12	Mean 40,000	PEB (N/A)	NED (6y)
	2			PEB (N/A)	NED (9y)
	3			PEB (N/A)	Recurrence after 1 y and DOD in 3 mo
Lacy et al. [17]	1	7	2,364	PEB (5)	NED (21 mo)
Terenziani et al. [18]	1	6	3,006	PEB (6)	NED (14 y)
	2	24	104,340	PEB (4)	NED (35 mo)
Rajagopal et al. [19]	1	17	80,892	PEB (6)	NED (5 y)
	2	18	2,139	PEB (4)	NED (13 y)
	3	23	87,680	PEB (6)	Local recurrence 4 mo later, NED (20 y)
	4	3	2,180	PEB (10)	Local recurrence 4 mo later, NED (24 y)
	5	23	> 10,000	PEB (8)	NED (25 y)
Ahsan et al. [20]	1	16	14,321	PEB (6)	NED (5 y)

PEB, cisplatinn, etoposide, bleomycin; NED, no evidence of disease; DOD, dead of disease; N/A, not available.

One patient (5.9%) in the present series developed interstitial lung disease after 1 cycle of PEB. Considering the potential pulmonary toxicity associated with bleomycin, PEB should be changed to non-bleomycin-containing regimens, such as PEV, when distinct pulmonary syndromes and pulmonary dysfunction are detected. Vaginal EST, similar to its more common ovarian counterpart, generally tends to relapse early, within 2 years following chemotherapy [13, 22].

Approximately half of all RMS cases in the genital tract arise in the vagina and most commonly in the first decade of life, while its less common cervical counterpart most commonly occurs in the second and third decades of life [8, 23, 24]. Most cases of vaginal RMS are the botryoid variant, which typically presents as a polypoid mass protruding from the vagina, resembling a bunch of grapes [8, 9, 25]. In our series, all 7 patients with vaginal RMS had the botryoid variant and all were younger than 3 years of age. If cervical and vaginal involvement is detected together, it is assumed a primary cervical malignancy [26]. Accordingly, we excluded the patient with embryonal RMS that had both cervical and vaginal involvement at the age of 144 months.

RMS is a highly aggressive tumor arising from the embryonal mesenchyme. The management and prognosis of RMS of the genital tract have changed dramatically over the last four decades. In the 1960s, radical surgical treatment, including pelvic exenteration, was the treatment of choice, and often resulted in a poor prognosis [27, 28]. Since then, multimodality therapy, including combination chemotherapy with or without radiotherapy with less radical surgery, was adopted and has significantly improved survival [29, 30]. At that time, a 3-year disease-free survival rate of 92% was demonstrated with chemotherapy and conservative surgery [8]. In the 1990s, there was a gradual shift toward more conservative surgeries, such as local excision, polypectomy, or cervical conization with or without chemotherapy [31, 32]. Four randomized trials completed by the IRSG and four clinical trials organized by SIOP have gradually changed the treatment protocols to more conservative approaches. Moreover, the prognosis was not compromised by a more conservative management [33, 34]. RMS is sensitive to chemotherapy, which has an important role in both local control, and eradication of potential micrometastatic disease. The most common chemotherapy regimens were VAC and IVA, which were chosen as mainstays of chemotherapy in US and European studies, respectively [35]. In our study, all 7 patients with vaginal botryoid RMS were treated with conservative surgery (6 underwent excisional biopsy and 1 underwent local excision) and combination chemotherapy. All 7 patients achieved a complete remission. Our results and the reports from the literature [33] demonstrate that conservative surgery with chemotherapy is effective for vaginal botryoid RMS. Local excision should be considered only when complete resection can be achieved without important functional impairment, while excisional biopsy is usually suitable for most cases as primary surgery. During chemotherapy, all cases should undergo a second evaluation with examination under anesthesia and resection of the residual mass, or tumor site biopsy.

Many patients with RMS may experience locoregional and/or metastatic recurrences, even if they have achieved complete remission with primary therapy. Dantonello et al. [36] analyzed recurrences in 1,164 patients with nonmetastatic RMS who achieved complete remission at the end of primary therapy. The results showed that 337 patients (29%) developed recurrences (mainly as locoregional recurrences), and the median time to recurrence was 1.43 years (range, 0.13 to 13.5 years) after diagnosis; only 2 patients developed metastatic recurrences more than 4 years after diagnosis. Our findings confirmed that recurrence must be expected in approximately every third patient with nonmetastatic RMS. In our series, 2 of the 7 patients (28.6%) developed locoregional recurrence at about 1 year after diagnosis at a median follow-up of 46 months (range, 23–102 months).

Our study has several limitations, including retrospective data collection, the small number of patients, single-institutional experience, and the bias caused by transferred patients. The first 2 patients with EST and the first patients with RMS in our series received chemotherapy without well-defined protocols. Two patients with EST who were initially treated at other hospitals received nonstandard treatments. Both of them underwent conservative surgery via laparotomy. Then, one received nonstandard chemotherapy, and the other one received too many cycles (10 cycles) of PEB. Moreover, this study covered nearly a 20-year period, during which time the treatment protocols for vaginal EST and RMS advanced.

In conclusion, to allow preservation of sexual and reproductive function, vaginal EST and botryoid RMS in childhood should be managed with conservative therapeutic strategies. PEB alone could be the first choice of treatment for children with vaginal EST. Conservative surgery in combination with chemotherapy should be considered the mainstay of treatment for vaginal botryoid RMS. Further large-scale studies with long-term follow-up are needed to confirm our results and to define the best treatment protocols for these malignancies.

## MATERIALS AND METHODS

### Patients

We reviewed retrospectively the records of all children diagnosed with vaginal EST and RMS at Peking Union Medical College Hospital between 1996 and 2015. Patients were treated at Peking Union Medical College Hospital or were referred to the hospital after initially being treated elsewhere. Vaginal masses were assessed with physical examination and imaging examinations. Serum tumor markers, including AFP, were tested in all cases. Patients with benign tumors, precancerous lesions, or metastatic malignancies were excluded. As a result, 18 patients with EST and 8 patients with RMS were identified. Then, two patients were excluded: 1 who had concurrent cervical and vaginal embryonal RMS at 144 months of age (for failing to confirm the original site), and 1 patient with EST who was initially treated at other hospitals without detailed treatment records.

The study protocol was approved by the Institutional Review Board of Peking Union Medical College Hospital.

### Clinicopathologic data

Clinicopathologic information, including patient age at diagnosis, clinical features, pathological findings, treatment modality, and follow-up data, were collected from the medical records and telephone calls. RMS was classified according to the ICR classification and staged according to the IRSG surgically based clinical grouping system [6, 7, 24], presented in Table 4. All eligible patients’ pathological results were reviewed and confirmed by two independent pathologists.

**Table 4:** Intergroup rhabdomyosarcoma study group (IRSG) clinical grouping classification

Group	Clinical findings
I	Localized disease, completely excised, no microscopic residual tumor
A	Confined to site of origin, completely resected
B	Infiltrating beyond site of origin, completely resected
II	Total gross resection
A	Gross resection with evidence of microscopic local residual disease
B	Regional disease with involved lymph nodes, completely resected with no microscopic residual tumor
C	Microscopic local and/or nodal residual disease
III	Incomplete resection or biopsy with gross residual disease
IV	Distant metastases
